# Anti-diabetic effect of black ginseng extract by augmentation of AMPK protein activity and upregulation of GLUT2 and GLUT4 expression in db/db mice

**DOI:** 10.1186/s12906-017-1839-4

**Published:** 2017-06-29

**Authors:** Ok-Hwa Kang, Mi-Yae Shon, Ryong Kong, Yun-Soo Seo, Tian Zhou, Do-Yeon Kim, Yeong-Soo Kim, Dong-Yeul Kwon

**Affiliations:** 1 Department of Oriental Pharmacy, College of Pharmacy and Wonkwang-Oriental Medicines Research Institute, Wonkwang University, 344-2 Sinyong-dong, Iksan, Jeonbuk 570-749 South Korea; 2 International Ginseng and Herb Research Institute, Geumsan, 312-804 South Korea; 3 Nakdonggang National Institute of Biological Resources, Sangju, 37242 South Korea

**Keywords:** Black ginseng, Anti-diabetic, AMPK, Glut

## Abstract

**Background:**

Black ginseng (*Panax ginseng* C. A. Meyer), three to nine times-steamed and dried ginseng, has biological and pharmacological activities. In this study, the anti-diabetic effects of the black ginseng ethanol extract (GBG05-FF) in typical type 2 diabetic model db/db mice were investigated.

**Methods:**

The effect of GBG05-FF in Type 2 diabetic mice was investigated by their blood analysis, biological mechanism analysis, and histological analysis.

**Results:**

The mice group treated with GBG05-FF showed decreased fasting blood glucose and glucose tolerance compared to that of the nontreated GBG05-FF group. In the blood analysis, GBG05-FF decreased main plasma parameter such as HbA1c, triglyceride, and total-cholesterol levels related to diabetes and improved the expression of genes and protein related to glucose homeostasis and glucose uptake in the liver and muscle. The histological analysis result shows that GBG05-FF decreased lipid accumulation in the liver and damage in the muscle. Moreover, GBG05-FF increased the phosphorylation of the AMPK in the liver and upregulated the expression of GLUT2 in liver and GLUT4 in muscle. Therefore, the mechanisms of GBG05-FF may be related to suppressing gluconeogenesis by activating AMPK in the liver and affecting glucose uptake in surrounding tissues via the upregulation of GLUT2 and GLUT4 expression.

**Conclusion:**

These findings provided a new insight into the anti-diabetic clinical applications of GBG05-FF and it might play an important role in the development of promising functional foods and drugs from the viewpoint of the chemical composition and biological activities.

## Background

Black ginseng (*Panax ginseng* C. A. Meyer), three to nine times-steamed and dried ginseng undergoes Maillard browning reaction. The biological and pharmacological activities of steam-processed ginseng are greater than that of nonsteamed ginseng. During the steaming and drying process, the percentage of main ingredients including saponin, ginsenosides, proteins, and phenolics is altered, because of newly produced active ingredients [1–4]. Black ginseng contains newly discovered ginsenosides and exhibits more potent biological activities than white and red ginseng [5]. Black ginseng possesses anticarcinogenesis, anti-diabetic, recovery from learning and memory damages, and antioxidative activities [6–9]. Previous studies were focused on verifying effectiveness of black ginseng on improvement of insulin resistibility or restoration of β-cell function [10]. In recent studies, we showed that supplementation with black ginseng extract improves diabetic parameters, thus it has a potential as a functional food for diabetes mellitus patients [11]. In this study, we focused on the regulation of hyperglycemia and hyperlipidemia caused by black ginseng extract.

Diabetes is a chronic metabolic disease that account for 3% of the world’s mortality, because of its complications; especially, disease incidence in South Korea is the highest out of all of the OECD countries. It is a metabolic disease caused by either the destruction of pancreatic β-cells (type 1 diabetes) or unresponsiveness to insulin (type 2 diabetes) [12]. In particular, type 2 diabetes is an alarming public health issue, because it causes a broad range of chronic diseases and metabolic syndromes such as cardiovascular disease, hypertension, non-alcoholic fatty liver disease (NAFLD), and obesity.

The homeostasis of blood glucose is maintained as a result of the action of various vital organs. Blood glucose influx is mediated by glucose transporter (GLUT). When the hyperglycemia broke out, plasma glucose entered the cytoplasm of hepatocytes through GLUT2 [13]. In contrast, the glucose uptake in adipose tissue and muscle is mostly mediated by GLUT4 and plays an important role in glucose utilization by insulin stimulation [14]. Hepatic glucose uptake and glucose production are the key parameters in glucose homeostasis. Adenosine monophosphate-activated protein kinase (AMPK), a serine/threonine kinase, has a critical function in this process, because it can suppress gluconeogenesis in the liver and promote glucose uptake in peripheral tissues [15]. Previous studies have found that the activation of AMPK signal pathways increased the insulin- and contraction-stimulated GLUT4 translocation in muscles [16].

In this study, the anti-diabetic effects of black ginseng extract (GBG05-FF) in typical type 2 diabetic model db/db mice were investigated. GLUT2 expression in the liver, GLUT4 expression in the skeletal muscle, AMPK protein levels in the liver and white fat was detected to validate the beneficial effects of GBG05-FF as an anti-diabetic agent and to clarify the mechanism of action.

## Methods

### Materials

3,3-Diaminobenzidine tetrahydrochloride (DAB) was purchased from Sigma-Aldrich (St Louis, MO, USA). Guinea pig antihuman insulin and rabbit antiglucagon were obtained from Millipore Corporation (Billerica, MA, USA). Glycated hemoglobin (HbA1c), triglyceride (TG) cholesterol (CHO), and oral glucose tolerance test (OGTT) were purchased from Bio-Rad variant II turbo (Bio-Rad Laboratories, Hercules, CA, USA). Glucose 6-phosphatase (G6pase), phosphoenolpyruvate carboxykinase (PEPCK), liver glycogen phosphorylase (LGP), carnitine palmitoyltransferase 1α (CPT1α), citrate synthase (CS), glucokinase (GK), mitochondrial medium-chain acyl-CoA dehydrogenase (MCAD), Glucose transporter 2 (GLUT2) and glucose transporter 4 (GLUT4), and β-actin oligonucleotide primers were purchased from Bioneer Corp. (Daejeon, Korea). Anti-β-actin, GLUT2, and GLUT4 antibodies were obtained from Santa Cruz Biotechnology (Santa Cruz, CA, USA). Anti-pThr172–5′ AMP-activated protein kinase (AMPK) and antiAMPK antibodies were purchased from Cell Signaling Technology (Beverly, MA, USA). Standard ginsenosides were purchased from Ambo institute (Daejeon, Republic of Korea).

### Preparation of GBG05-FF

GBG05-FF was made by the business of master-work for geumsan ginseng through International ginseng and Herb Research Institute, it was studied [11]. Briefly, 5-Year old Korean white ginseng (*Panax ginseng* C.A. Meyer) was purchased from a local ginseng center (Geumsan, Korea). The black ginseng was prepared from white ginseng by repeated steaming at 95 °C for 6 h and drying at 60 °C. The dried black ginseng samples were extracted twice with 10 volumes of 70% ethanol at 80 °C for 8 h, using a Soxhlet extractor with a heating mantle. The extracts were subsequently filtered and lyophilized. The extraction yields of black ginseng was 25%. Black ginseng extract was designated as GBG05-FF and used for the research.

### Analytical methods

**Fig. 1:**
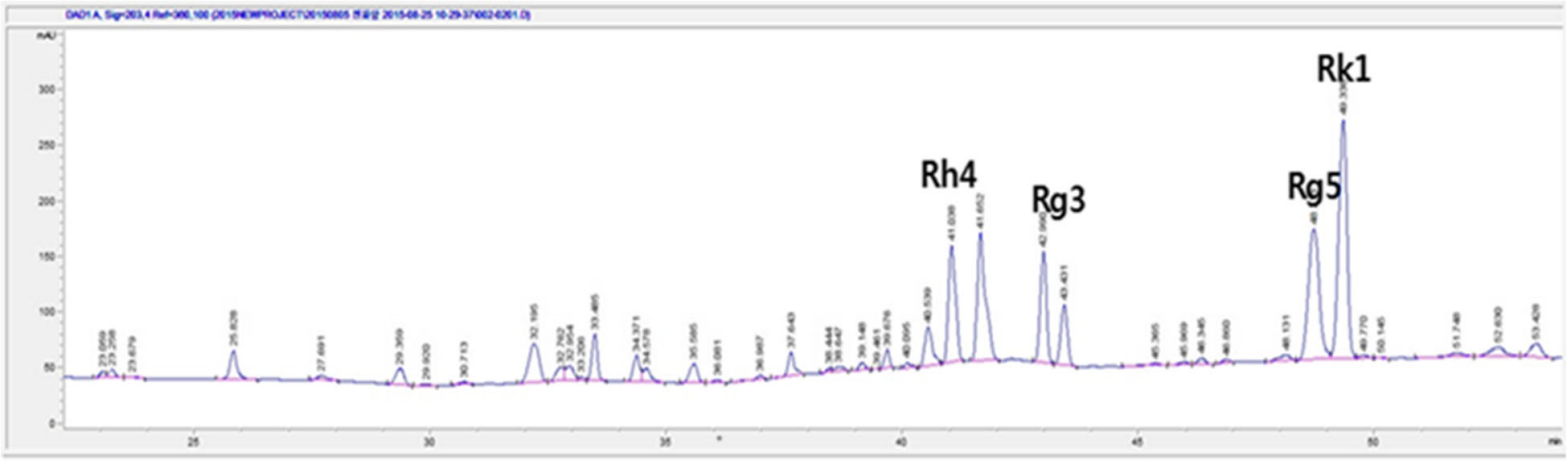
HPLC chromatograns of ginsenosides in GBG05-FF

GBG05-FF sample was extracted with an equal volume of n-butanol. The n-butanol fraction was then evaporated to dryness, and 50% methanol was added. HPLC system (Santa Clara, CA, USA) equipped with a Poroshell 120 EC-C18 column (3.0 mm × 50 mm, 2.7 μm, Agilent) was used at a detection wavelength of 203 nm. Water (A) and acetonitrile (B) were kept at 35 °C and used as the mobile phase at 0.8 ml/min with following gradient: 0–2 min, 10% B; 2–4 min, 11% B; 4–9 min, 13% B; 9–19 min, 21% B; 19–26 min, 28% B; 26–28 min, 28% B; 28–32 min, 32% B; 32–34 min, 32% B; 34–40 min, 45% B; 40–42 min, 45% B; 42–50 min, 55% B; 50–55 min, 55% B; 55–55.01 min, 10% B; 55.01–62 min, 10% B. To investigate the stability of the GBG05-FF water decoction, the same extraction step was repeated to obtain GBG05-FF and then the berberine hydrochloride content in GBG05-FF was detected by HPLC analysis at a detection wavelength of 203 nm with a C18 column (Fig. 1).

### Experimental mouse model of diabetes

All the animal experiments were performed in accordance with the guidelines established by the Animal Ethics Committee of Wonkwang University, and this experiment was approved by the committee (Approval No. WKU15–101). To evaluate the effect of GBG05-FF on fasting blood glucose levels, 30 C57BKS (db/db) mice (20–25 g, 4 weeks, male, BKS.Cg − +Lepr^db^/+Lepr^db^) and 10 C57BKS mice (20–25 g, four weeks, male, BKS.Cg-m+/+Lepr^db^) were used. All the animals were purchased from SAMTACO (Osan, Korea). All the experiments in this study were conducted for four weeks. The mice were given free access to water and kept at a constant room temperature under a 12/12–hour light/dark cycle. All the mice were acclimatized for an adaptation period of seven days before the experiment, fed standard pellet chow, and given fresh water ad libitum. A total of 40 mice were randomly divided into four groups (ten mice per group): Group I (C57BKS group, Normal), Group II (C57BKS -db/db group, Control), Group III (C57BKS -db/db mice were fed a dietary supplement of 300 mg/kg of GBG05-FF, GBG05-FF 300), and Group IV (C57BKS-db/db mice fed a dietary supplement of 900 mg/kg of GBG05-FF, GBG05-FF 900). The weights were checked every week. Water and feed were always available. After four weeks, all the mice were sacrificed to collect blood and remove the liver and muscle.

### Measurement of fasting blood glucose level

Fasting blood glucose levels were checked from blood samples taken from the tail vein of each mouse every week. To determine fasting blood glucose levels, mice were deprived of their diet for 12 h, but allowed free access to drinking water. Blood glucose levels were checked by using Acuu-Check (Roche Diagnostics Korea co., Ltd., Seoul, Korea).

### Measurement of oral glucose tolerance test (OGTT)

After GBG05-FF administering for four weeks, the OGTT were performed. Briefly, a normal control group, diabetic control group, and GBG05-FF-fed groups (GBG05-FF 300 and GBG05-FF 900) were deprived of their diet, but allowed access to drinking water. After fasting for 16 h, blood was drawn from the mouse tail veins and glucose levels were determined by using Acuu-Check of all the mice. Immediately after the blood check, glucose (0.2 g/100 g body weight) was orally administered to all mice. Blood glucose levels were checked at the indicated time intervals (0, 30, 60, 90, and 120 min).

### Glycated hemoglobin, TG, and total cholesterol assays in the serum

After administering GBG05-FF for four weeks, the mice were sacrificed and the blood collected was used to evaluate HbA1c, TG, and total CHO levels. HbA1c levels were measured by a Bio-Rad variant II turbo (Bio-Rad Laboratories, Hercules, CA, USA). Plasma TG and total CHO levels were measured using an Automated chemical analyzer (Roche Diagnostics, Mannheim, Germany).

### Histopathological analysis of liver and mucsle

The liver and muscle tissues were fixed in 4% formalin over 24 h. The fixed tissues were processed for paraffin embedding. The sections were counterstained with hematoxylin and eosin. To observe collagen content, picrosirius red staining was performed by using a picrosirius red staining kit (Polysciences, Inc. Warrington). The sections of muscle were deparaffinized and rinsed with distilled water. The washed sections were soaked in picrosirius red solution A for 2 min. The sections were rinsed again with distilled water, soaked in picrosirius red solution B for 60 min and picrosirius red solution C for 2 min, immersed in 70% alcohol for 45 s, dehydrated, cleared, and mounted. Image acquisition was performed using an optical microscope (Nikon Eclipse 80i; Tokyo, Japan) with a magnifying power of 100 and 200.

### Quantitative reverse transcription polymerase chain reaction (RT-PCR) analysis

Total RNA was isolated from the muscles and liver in mice using an RNeasy Mini kit (iNtRon, Seongnam, Korea), and complementary deoxyribonucleic acid (cDNA) was synthesized using a the QuantiTect Reverse Transcription kit (Qiagen, Seoul, Korea) according to the manufacturer’s instructions. Real-time PCR was performed in triplicate using power SYBR® Green PCR master mix (iNtRon, Seongnam, Korea) in a StepOnePlus Real-Time RT-PCR System. The expression levels of the target genes relative to that of the endogenous reference gene β-actin were calculated using the delta cycle threshold method using StepOne software v2.3 (Applied Biosystems, Foster City, CA, USA). The primer sequences are listed in Table 1.

**Table 1:** Primers for quantitative real-time polymerase chain reaction analysis

	Forward	Reverse
G6Pase	ATGACTTTGGGATCCAGTCG	TGGAACCAGATGGGAAAGAG
PEPCK	CTGGCACCTCAGTGAAGACA	TCGATGCCTTCCCAGTAAAC
LGP	CCAGAGTGCTCTACCCCAAT	CCACAAAGTACTCCTGTTTCAGC
GS	GACACTGAGCAGGGCTTTT	GGGCCTGGGATACTTAAAGC
CPT1α	ACCCTGAGGCATCTATTGACAG	ATGACATACTCCCACAGATGGC
MCAD	GGCAAATGCCTGTGATTCTT	CCATTGCGATCTTGAAACCT
GK	TCCCTGTAAGGCACGAAGACAT	ATTGCCACCACATCCATCTCA
CS	TGCCCACACAAGCCATTTG	CTGACACGTCTTTGCCAACTT
SIRT4	CGCTGCTCAAGATCCCTAAG	GCGACACAGCTACTCCATCA
SIRT6	GGCTACGTGGATGAGGTGAT	GGCTCAGCCTTGAGTGCTAC
GLUT2	CTGCTCTTCTGTCCAGAAAGC	TGGTGACATCCTCAGTTCCTC
GLUT4	TCGTCATTGGCATTCTGGTTG	AGCTCGTTCTACTAAGAGCAC

### Western blot analysis

Protein expression was assessed by Western blot analysis according to the standard procedures. Each liver and muscle were homogenized in RIPA lysis buffer (ATTO Corp., Tokyo, Japan) on ice. The homogenates were centrifuged (13,000 rpm, 10 min, 4 °C), and the protein concentrations in the supernatant were determined using the Bio-Rad protein assay reagent (Bio-Rad Laboratories, Hercules, CA, USA) according to the manufacturer’s instructions. Equal amounts of protein (20 μg) were subjected to sodium dodecyl sulfate-polyacrylamide gel electrophoresis and transferred to a polyvinylidene membrane (Millipore, Bedford, MA, USA). The membrane was blocked for >1 h with 5% skim milk in Tris-buffered saline buffer (150 mM NaCl and 20 mM Tris-HCl, pH 7.4) with 0.05% Tween 20. The membrane was incubated with primary antibodies for 18 h, washed with tris-buffered saline containing Tween 20, and incubated with anti-mouse or anti-rabbit immunoglobulin G horseradish peroxidase-conjugated secondary antibodies combined with primary antibody of target protein. The proteins were supplemented with the ECL prime Western blotting detection reagents (GE Healthcare, Parsippany, NJ, USA), and ImageQuant LAS 4000 Mini Biomolecular Imager (GE Healthcare, Parsippany, NJ, USA) was used to evaluate the bands, which were quantified by Image j.

### Statistical analysis

All the experimental results were compared by one-way analysis of variance (ANOVA) using the Statistical Package for the Social Sciences (SPSS, ver. 22.0, SPSS Inc. Chicago, IL, USA). The data are expressed as means ± SEM. Group means were considered to be significantly different at *p* < 0.05, as determined by the technique of protective least significant difference.

## Results

### HPLC analysis of GBG05-FF

The contents of minor ginsenoside Rh4, Rk1 and Rg5 in BGE (GBG05-FF) were 1.93, 6.90, and 11.74 mg/g, respectively. GBG05-FF contained ginsenosides such as ginsenoside Rg5, Rk1, and Rh4. These unique ginsenosides are nonexistent in white ginseng. The major ginsenosides including Rb1, Rb2, Rc, and Rd. in white ginseng can be transformed to the minor ginsenoside Rg3. This ginsenoside may also be converted to Rg5 and Rk1 by heating (Table 2).

**Table 2:** Ginsenoside contents in GBG05-FF

Ginsenosides (mg/g)		
Rh4	Rg5	Rk1
1.93±0.18	11.74±0.27	6.9±0.08

### Effect of GBG05-FF on fasting glucose levels and glucose intolerance in type-2 diabetes model

**Fig. 2:**
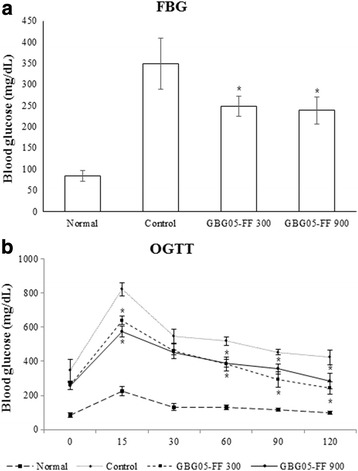
Effect of GBG05-FF on fasting blood glucose levels (**a**) and glucose tolerance (**b**) in mice. Normal: dbh, Control: db/db, GBG05-FF 300: db/db fed the GBG05-FF 300 mg/kg, and GBG05-FF 900: db/db fed the GBG05-FF 900 mg/kg. Each value represents the mean ± SEM (*n* = 10 per group). * *P* < 0.05: Significant difference vs. control group

To confirm the effect of GBG05-FF on the development of diabetes, fasting blood glucose levels and glucose tolerance were investigated in Type 2 diabetic db/db mice. Before GBG05-FF was administered to mice, the zero-time fasting blood glucose levels were not significantly different among the groups. During four weeks of GBG05-FF administering, GBG05-FF suppressed the increase in the fasting blood glucose level in Type 2 diabetic db/db mice (Fig. 2). Moreover, after GBG05-FF administration for four weeks, OGTT was performed. The result of glucose tolerance in the mice group administered with GBG05-FF showed decrease in glucose level compared to that of the control group (Fig. 2). However, the result showed no dose dependent effect both on fasting glucose levels and glucose tolerance.

### Effect of GBG05-FF on plasma parameters related to diabetes

**Fig. 3:**
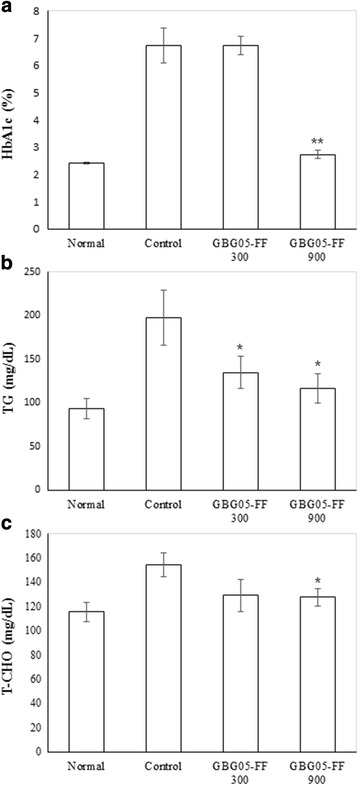
Effect of GBG05-FF on Glycated hemoglobin (**a**), Triglyceride (**b**) and Total-cholesterol (**c**) in blood serum of mice. Normal: dbh, Control: db/db, GBG05-FF 300: db/db fed the GBG05-FF 300 mg/kg, and GBG05-FF 900: db/db fed the GBG05-FF 900 mg/kg. Each value represents the mean ± SEM (*n* = 10 per group). * *P* < 0.05, ** *P* < 0.01: Significant difference vs. control group

To verify the effect of GBG05-FF in plasma parameters, blood samples were collected from mice after experiment during four weeks. The blood analysis results showed that the TG and total CHO decreased in a dose-dependent manner, but HbA1c levels decreased only for the group administered to GBG05-FF 900 mg/kg with 900 mg/kg GBG05-FF (Fig. 3).

### Effect of GBG05-FF on the expressions of genes related to glucose homeostasis in muscle and liver of type 2 diabetic db/db mice

To prove the effect of GBG05-FF in glucose homeostasis mechanism of diabetic mice, quantitative real-time PCR analysis was performed. The results showed that GBG05-FF suppressed the expression of G6Pase, PEPCK, and LGP in the liver tissue, whereas GBG05-FF increased the expression of GS in the liver tissue and the expression of CPT1α, CS, GK, and MCAD in the muscle tissue.

### Histological analysis on effect of GBG05-FF in muscle and liver tissue

**Fig. 4:**
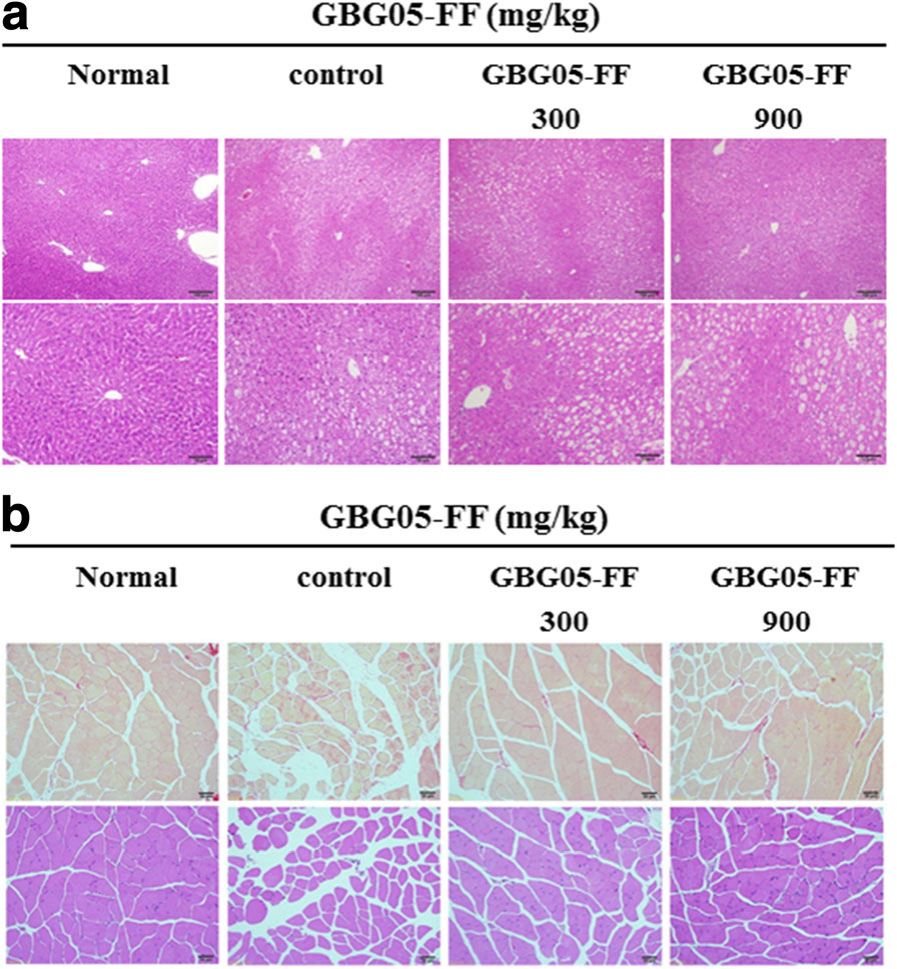
Effects of GBG05-FF on histological change in liver and in muscle. The upper macrophotographs are liver magnified to 100 times their actual size, and the lower macrophotographs are liver magnified to 200 times their actual size. (**a**) Effects of GBG05-FF on histological change in muscle. The upper macrophotographs are Picrosirius red staining, and the lower macrophotographs are H & E staining (**b**). Normal: dbh, Control: db/db, GBG05-FF 300: db/db fed the GBG05-FF 300 mg/kg, and GBG05-FF 900: db/db fed the GBG05-FF 900 mg/kg

To investigate the morphological changes of the liver and muscle, histological analysis was performed. The liver of db/db group not treated with GBG05-FF showed lipid accumulation. However, the liver of db/db group treated GBG05-FF showed a significant decrease on the lipid accumulation (Fig. 4a). The result of the histological analysis of muscle showed that muscle of the non-treated GBG05-FF group significantly decreased myofibers compared to that of the normal group, whereas the mice group treated with GBG05-FF showed the restoration of myofibers by GBG05-FF. Moreover, through picrosirius red staining, we confirmed that GBG05-FF decreased muscle damage by Type 2 diabetes (Fig. 4b).

### Effect of GBG05-FF on expresstion gene and protein levels and glucose uptake in liver and muscle of type 2 diabetic db/db mice

**Fig. 5:**
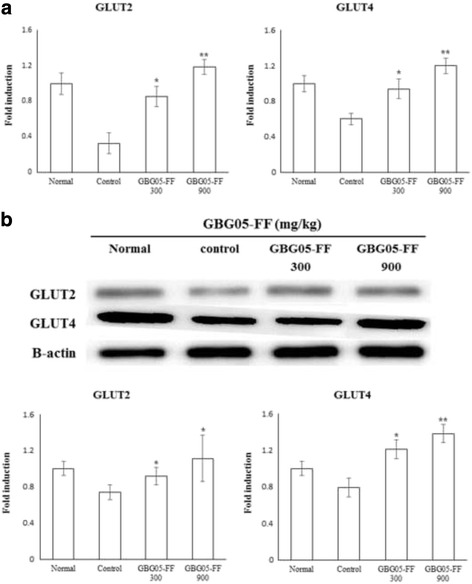
Effects of GBG05-FF on the gene expression and the protein expression of GLUT2 in liver (**a**), and the gene expression and protein expression of GLUT4 in muscle (**b**) of mice. Normal: dbh, Control: db/db, GBG05-FF 300: db/db fed the GBG05-FF 300 mg/kg, and GBG05-FF 900: db/db fed the GBG05-FF 900 mg/kg. Each value represents the mean ± SEM (*n* = 10 per group) in three independent experiments. *P < 0.05, ** *P* < 0.01: Significant difference vs. control group

To confirm the effect of GBG05-FF in glucose uptake mechanism of diabetic mice, quantitative real-time PCR and western blotting analysis were performed. The results show that GBG05-FF increased gene expression of both GLUT2 and GLUT4. Moreover, GLUT2 in the liver and GLUT4 were upregulated by GBG05-FF in the muscles (Fig. 5).

### Effect of GBG05-FF on AMPK phosphorylation in type 2 diabetic db/db mice

**Fig. 6:**
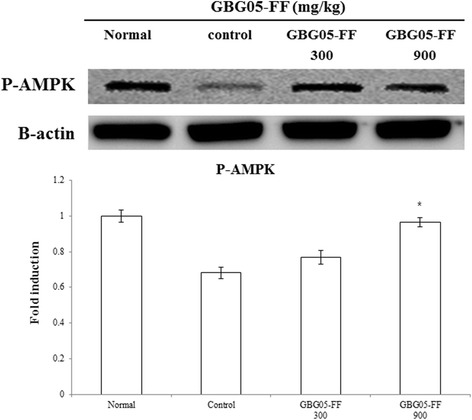
Effects of GBG05-FF on the phosphorylation of AMPK in livers of mice. Normal: dbh, C2ntrol: db/db, GBG05-FF 300: db/db fed the GBG05-FF 300 mg/kg, and GBG05-FF 900: db/db fed the GBG05-FF 900 mg/kg. Each value represents the mean ± SEM (*n* = 10 per group) in three independent experiments. * *P* < 0.05, ** *P* < 0.01: Significant difference vs. control group

To confirm the effect of GBG05-FF on AMPK phosphorylation in the liver of diabetic mice, western blotting analysis was performed. The result showed that phosphorylation of AMPK were increased by GBG05-FF in the liver (Fig. 6).

## Discussion

In this study, we demonstrated that GBG05-FF decreased hyperglycemia and hyperlipidemia by reducing the blood glucose levels and clarified the mechanism of action in type 2 diabetic C57BKS -db/db mice. Recently, there has been a growing interest in hypoglycemic agents from natural products, in particular, those derived from plants [17]. Therefore, searching for better agents from herbs or natural products that can be used to treat diabetes is necessary.

Ginseng is a well-characterized medicinal herb listed in the classic oriental herbal. Ginseng has diverse pharmacological and therapeutic properties such as immune function, cancer, and cardiovascular diseases, and has shown improved physical, cognitive, and sexual performance [18]. Moreover, many studies have reported that dietary ginseng saponins may have a beneficial effect on hyperlipidemia and obesity as well as reducing total serum cholesterol level [19, 20]. In Asia, there are a variety of commercial ginseng products including white, red, and black ginseng. Especially, black ginseng is produced by a repeated steaming and drying process from three to nine times. Black ginseng possesses better biological activity inducing anti-inflammatory, anti-cancer, anti-stress, and free radical scavenging effects than those of red ginseng [21, 22]. Although black ginseng is pharmacologically effective, the anti-diabetic effect of GBG05-FF has not been examined yet.

First of all, to investigate the major compound that has the effect to lower glucose levels, we were performed using several compounds contained in GBG05-FF profiled from HPLC assay (Fig. 1). GBG05-FF contained ginsenosides such as ginsenoside Rg5, Rk1, and Rh4. These unique ginsenosides are nonexistent in white ginseng. The major ginsenosides including Rb1, Rb2, Rc, and Rd. in white ginseng can be transformed to the minor ginsenoside Rg3. This ginsenoside may also be converted to Rg5 and Rk1 by heating [23]. In particular, delycosylated minor ginsenosides have more pharmaceutical activities than major ginsenosides [24].

**Fig. 7:**
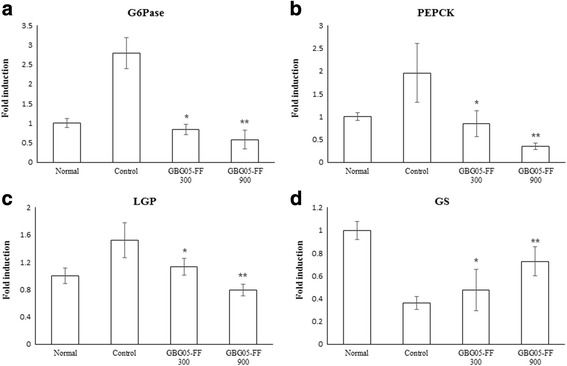
Effects of GBG05-FF on the expression of G6Pase (**a**), PEPCK (**b**), LGP (**c**) and GS (**d**) in the liver of mice. Normal: dbh, Control: db/db, GBG05-FF 300: db/db fed the GBG05-FF 300 mg/kg, and GBG05-FF 900: db/db fed the GBG05-FF 900 mg/kg. Each value represents the mean ± SEM (*n* = 10 per group) in three independent experiments. *P < 0.05, **P < 0.01: Significant difference vs. control group

**Fig. 8:**
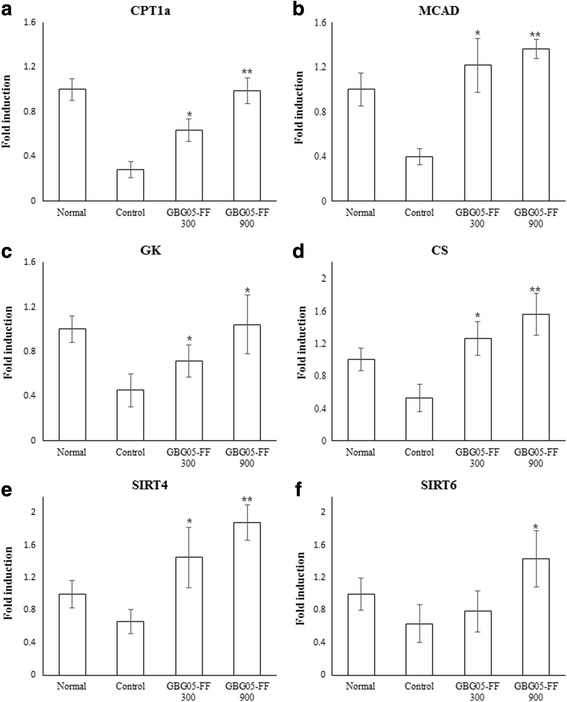
Effects of GBG05-FF on the expression of CPT1α (**a**), MCAD (**b**), GK (**c**), CS (**d**), SIRT4 (**e**) and SIRT6 (**f**) in the muscle of mice. Normal: dbh, Control: db/db, GBG05-FF 300: db/db fed the GBG05-FF 300 mg/kg, and GBG05-FF 900: db/db fed the GBG05-FF 900 mg/kg. Each value represents the mean ± SEM (*n* = 10 per group) in three independent experiments. * *P* < 0.05, ** *P* < 0.01: Significant difference vs. control group

Hepatic glucose uptake and glucose production in peripheral tissues are highly important in glucose homeostasis of the body. We proposed that GBG05-FF is effective in inhibiting hepatic gluconeogenesis and promoting glucose uptake of peripheral tissue. Elevated hepatic glucose production is a major cause of hyperglycemia in Type 2 diabetes. G6pase, PEPCK, liver glycogen phosphorylase (LGP), and glycogen synthase (GS) enzymes are important factors related to gluconeogenesis and glycogenolysis in the liver [25]. CPT1 is significant for fatty acid oxidation in the liver [26]. Mitochondrial medium-chain acyl-CoA dehydrogenase (MCAD) are significant for β-oxidation. Glucokinase (GK) and citrate synthase (CS) are related to glucose oxidation. [27]. Our results suggest that GBG05-FF suppressed the expression of G6Pase, PEPCK, and LGP in the liver tissue (Fig. 7), whereas it increased the expression of GS in the liver tissue and the expression of CPT1α, CS, GK, and MCAD in the muscle tissue. In addition, GBG05-FF increased the expression of SIRT4 and SIRT6, which belong to the sirtuin family of proteins that regulate insulin secretion and glucose homeostasis (Figs. 7 and 8) [28, 29]. Taken together, our results suggest that GBG05-FF significantly decreased the lipid accumulation and muscle damage by Type 2 diabetes (Fig. 4).

AMPK is a phylogenetically conserved serine/threonine protein kinase that has been proposed to act as a target for the prevention of the metabolic syndrome, characterized in hypertension, obesity, dislipidemia and insulin resistance, eventually leading to type 2 diabetes. In particular, AMPK is an *αβγ* heterotrimer, has a main function in regulating gluconeogenesis, and glucose uptake in surrounding tissues and is mediated by GLUT4 [30]. AMPK phosphorylation can directly phosphorylate CREB-regulated transcription coactivator 2 on Ser171, which would be antagonistic to the induction of gluconeogenic genes [31]. β-Cells are designed to sense blood glucose and other secretagogues to adjust insulin secretion according to the needs of the organism [32]. Glucose enters β-cells by facilitating diffusion through the glucose transporter (GLUT2 in rodents; mainly GLUT1 in humans) and is retained inside the cell through its phosphorylation by glucokinase [12]. This glucose sensor mechanism regulates glycolytic flux to the plasma glucose concentration in hepatocytes [33]. In contrast, glucose uptake in surrounding tissues is mediated by GLUT4 [30], which is the major glucose transporter of muscle and adipose tissues and facilitates intracellular glucose delivery from extracellular milieu, thus augmenting glucose uptake. GLUT4 mRNA and protein content can decrease in the peripheral tissues and may be one of the reasons for insulin resistance [34]. To evaluate the effect of GBG05-FF on GLUT2 and GLUT4 expression in the surrounding tissue and AMPK protein in the hepatic tissue, quantitative RT-PCR and Western blot analysis were used in our study. As shown in Fig. 5, GBG05-FF significantly increased GLUT2 and GLUT4 mRNA and protein level compared to the control group. As expected, the data shows that GBG05-FF significantly induced AMPK phosphorylation in hepatic tissues and increased the expression of GLUT2 in liver and GLUT4 in skeletal muscle and abdominal fat of db/db mice compared to the control group. These results indicate that GBG05-FF effectively inhibited AMPK-regulated gluconeogenesis and enhanced glucose uptake in surrounding tissues by upregulating the expression of GLUT2 and GLUT4.

## Conclusion

We have proved in this study that GBG05-FF regulates hyperglycemia and hyperlipidemia in mice. And the results confirmed that GBG05-FF possessed anti-diabetic activities in db/db mice. As a result of its fewer side effects, especially GBG05-FF has high potential in regulating glucose metabolism. Our data prove that GBG05-FF has multiple targets in the hypoglycemic mechanism, such as suppressing gluconeogenesis through AMPK activation in the liver and affecting glucose uptake of surrounding tissues by upregulating the expression of GLUT2 in liver and GLUT4 in muscle. These findings suggest that black ginsengs might play an important role in the development of promising functional foods and drugs from the viewpoint of the chemical composition and biological activities of black ginsengs with a distinction from those of white and red ginsengs.

## Abbreviations

AMPK: 5’adenosine monophosphate-activated protein kinase
ANOVA: One-way analysis of variance
CHO: Cholesterol
CPT1α: Carnitine palmitoyltransferase 1α
CS: Citrate synthase
DAB: 3,3-Diaminobenzidine tetrahydrochloride
G6pase: Glucose 6-phospatase
GBG05-FF: Black ginseng ethanol extract
GK: Glucokinase
GLUT: Glucose transporter
HbA1c: Glycated hemoglobin
LGP: Liver glycogen phosphorylase
MCAD: Mitochondrial medium-chain acyl-CoA dehydrogenase
NAFLD: Nonalcohlic fatty liver disease
OGTT: Oral glucose tolerance test
PEPCK: Phosphoenolpyruvate carboxykinase
RT-PCR: Reverse transcription polymerase chain reaction
SPSS: Statistical Package for the Social Sciences
TG: Triglyceride
